# Assessment protocol for acquired apraxia of speech

**DOI:** 10.1590/2317-1782/20232022251en

**Published:** 2023-10-13

**Authors:** Beatriz Maurer Costa, Cláudia Regina Brescancini, Karin Zazo Ortiz

**Affiliations:** 1 Departamento de Fonoaudiologia, Escola Paulista de Medicina, Universidade Federal de São Paulo - UNIFESP - São Paulo (SP), Brasil.; 2 Programa de Pós-graduação em Letras, Escola de Humanidades, Pontifícia Universidade Católica do Rio Grande do Sul - PUCRS - Porto Alegre (RS), Brasil.

**Keywords:** Apraxia, Articulation Disorders, Protocol, Symptom Assessment, Diagnosis

## Abstract

**Purpose:**

To develop an assessment protocol for speech motor planning with phonologically balanced stimuli for Brazilian Portuguese, including all necessary variables for this diagnosis.

**Methods:**

Three stages were carried out: In the first, word lists were built with the main criterion being syllabic and accentual patterns. From the survey conducted in Stage 1, the words that composed the first version of the protocol lists in Stage 2 were selected, and grouped into two fundamental tasks for diagnosing acquired apraxia of speech (AOS): repetition and Reading Aloud (RA). In Stage 3, the occurrence of words was investigated using the Brazilian Corpus (PUC-SP) - Linguateca database, and a statistical analysis was performed to verify if the repetition and RA lists were balanced in terms of the occurrences. Thus, the lists were distributed in quartiles and submitted to both descriptive and bivariate analyses. A significance level of 5% (p<0.05) was adopted.

**Results:**

After completion of all stages, the words that composed the lists of the repetition and RA tasks were obtained. Finally, other tasks considered essential for the assessment of AOS, such as diadochokinetic rates and the board for spontaneous oral emission, were then added to the protocol.

**Conclusion:**

The developed protocol contains the tasks considered standard for the assessment of AOS according to the international literature, which makes this instrument important for diagnosing this disorder in speakers of Brazilian Portuguese.

## INTRODUCTION

Acquired apraxia of speech (AOS) is described as “[...] a disorder of speech motor planning primarily manifested by articulation errors”^(1)^. It can occur as a result of various neurological insults, such as stroke, traumatic brain injury, tumors invading the central nervous system, and neurodegenerative diseases. AOS often coexists with aphasia^(2)^, which hinders the differentiation between phonological (aphasia) and phonetic (apraxia) manifestations.

Among the characteristics of AOS, there are the repetition of phonemes and syllables, self-correction, and articulatory rehearsal. Manifestations such as vowel prolongation, increased inter-syllabic distance, phonemic distortion, and intrusive *schwa* are more directly associated with AOS^(2)^. Substitution, omission, and addition—common in conduction aphasia—can also be observed in patients with apraxia^(2)^. Apraxia can also be characterized in prosody by slower and more hesitant speech with changes in rhythm and intonation due to issues in articulatory planning^(2,3)^.

In addition to describing manifestations, it is possible and recommended to use linguistic models of speech production to analyze types of errors to understand the processes involved in speech disorders, and thus characterize them more comprehensively. Currently, the most widely accepted speech production model^(4)^ identifies four levels of processing: the first is the pre-motor level, also known as linguistic-symbolic planning, where phoneme selection and application of linguistic rules occur; the second level is motor planning, which is responsible for motor memories and the temporal-spatial ordering of the phonemes to be produced; the third level is motor programming, where the appropriate sequence of muscles used to produce the phonemes is selected; in the fourth level, there is the execution of the speech sequence produced by the articulators involved in the action.

According to this model, signs of aphasia appear as a deficit in the linguistic-symbolic planning stage, whereas signs of apraxia appear in the motor planning and programming stages.

Some characteristics are typical while others are exclusive to AOS. Sound distortion, vowel and consonant prolongation, and prolonged duration of intersegments are exclusive to AOS^(2,3)^. These characteristics are more evident in polysyllabic words^(5)^. Even with the publication of the speech processing model and the observation of motor planning-related characteristics, differentiating between phonological and phonetic errors remains a highly complex task^(2,6)^.

With this change in view and interpretation of the speech and language interface based on speech processing models, new protocols have been developed aiming to make the assessment process more effective in differentiating language (aphasia) from speech (apraxia) disorders.

Internationally, the Apraxia Battery for Adults, ABA-2^(7)^, stands out, which includes word list tasks to assess the presence and measure the severity of the patient’s AOS. This is done through the Errors on Words of Increasing Length (E_WIL), a measure that relates errors to the length of the word^(2)^. The Apraxia of Speech Rating Scale (ASRS) is a perceptual scale that, like the ABA-2, is based on the evaluator’s auditory perception^(8)^. These protocols emphasize the accurate control of the stimuli used with variables currently considered fundamental for the diagnosis of AOS, including phonological balance. As the analysis is subjective, some protocols aim to reduce subjectivity, such as the Word Syllable Duration (WSD), which calculates the word production time divided by the number of syllables produced using acoustic measures^(9)^, and the Pairwise Variability Index (PVI), which uses words, phrases, and sentences to calculate the difference between stressed and unstressed syllables based on duration, fundamental frequency, and intensity^(10)^. Nevertheless, all protocols include linguistic stimuli with variables that interfere with motor planning, such as syllable frequency and structure, word stress position, phonological balance, word frequency and length, and phonotactic probability^(2,3,5,8)^. Moreover, the use of spontaneous speech tasks and diadochokinetic rates is recommended^(8)^.

In Brazil, there is a scarcity of objective procedures for the clinical assessment of AOS. The only published protocol for the clinical evaluation of this condition^(11)^ lacks the control of linguistic variables necessary for an accurate appraisal of motor planning, as well as tasks currently considered fundamental for this diagnosis.

Given the change in understanding of speech motor production arising from new processing models and the presence of new tools, there is a need to develop a protocol for Brazilian Portuguese that includes variables that interfere with motor speech planning and production.

Therefore, this study aims to develop an assessment protocol for AOS with important diagnostic variables and phonologically balanced stimuli for Brazilian Portuguese.

## METHODS

This study was conducted at the Department of Speech-Language Pathology at the Escola Paulista de Medicina (EPM) of the Universidade Federal de São Paulo (UNIFESP).

The protocol was developed in three stages:

In the first stage, word lists were built considering content words, specifically nouns and adjectives. In addition to proper nouns, inflected or infinitive verbs, functional words, words with context for epenthesis, and words with specialized meanings were excluded. In this stage, the main criteria for constructing the lists were syllabic and accentual patterns. Words that encompassed the syllabic patterns of the Portuguese language, from the canonical CV to the more complex CCVCC, with accentual alternation, were considered. Dissyllabic and trisyllabic words formed solely by the CV syllabic pattern, namely CV.CV and CV.CV.CV, were included in all accentual positions.

The following syllabic patterns were also included: CCV in a tonic position at the beginning of a word; in both tonic and atonic positions in the middle of a word; CVC in both tonic and atonic syllables; CVCC in an atonic syllable for /lS/ and /RS/ codas, and a tonic syllable for /NS/; CVCC in both tonic and atonic syllables; CVG (consonant vowel glide) in both atonic and tonic syllables, starting from the phonological diphthongs /ai/, /ei/, /oi/, and /ui/.

Based on the survey conducted in Stage 1, the words that composed the initial version of the list were selected. A second stage was necessary because of the excessive number of words, which would preclude the application of the protocol. The need for the protocol to consist of words with the same level of complexity in both the Repetition and Reading Aloud (RA) tasks was also considered. To this end, a controlled distribution of items between the two tasks was performed, so that when considering all the oral vowels of all items in the protocol, the words in the Repetition Task and the RA Task had the same proportion of each vowel. The same applied to the distribution of words regarding the voiced and voiceless phonemes, which were controlled.

During this distribution, to make the lists identical concerning the predetermined variables, three words would have to be added. These words would correspond to those that have the **ls** and **rs** codas, namely solstice (*so* ***ls*** *tício*) and perspicacious (*pe* ***rs*** *picaz*), and the one with the complex **fl** onset, specifically the word superfluous (*supér* ***fl*** *uo*). For the CV/lS/ syllable, there was only the word “*so* ***ls*** *tício*” in Portuguese, given the adopted criteria. Since there is no corresponding word in terms of articulatory complexity for both tasks, this word was excluded.

For the CV/RS/ syllable, the nouns found in the language were perspective (*pe* ***rs*** *pectiva*), scrutiny (*pe* ***rs*** *crutação*), superstition (*supe* ***rs*** *tição*) and interstice (*inte* ***rs*** *tício*). The words “*supe* ***rs*** *tição*” and “*inte* ***rs*** *tício*” were chosen to remain on the list.

On the other hand, the word superfluous (*supérfluo*), present in the first word list because of its complex onset in an atonic (post-tonic) syllable, lacked a corresponding word in the RA list because the only occurrence of this pattern occurs in the word septiform (*setênfluo*), which is not even recorded in the Brazilian Corpus database. As an alternative, the word confluent (*confluído*) was considered. However, they did not match in terms of occurrence frequency; therefore, this word was not included in the list.

The complexity of the syllabic and accentual pattern, as well as the phonetic and phonological variables, were controlled because they allow for an accurate analysis of errors, including the analysis of prosody, in its emphasis aspect Additionally, the duration of words can contribute to the analysis of speech production speed.

In addition to the criteria related to the complexity of the syllabic and accentual patterns, as well as the phonetic and phonological variables, a third stage was necessary to control the frequency variable of the words. The selection of the vocabulary in this stage was based on the number of occurrences found in the Brazilian Corpus (PUC-SP), using the *Linguateca* tool. To this end, all occurrences of the words pre-selected in Stages 1 and 2 were identified and then statistically analyzed using quartiles.

Thus, the lists underwent descriptive (by calculating summary measures) and bivariate analysis using Spearman’s rank correlation coefficient (*ρ*). All analyses were processed using the *R* 4.2.1 software. A significance level of 5% (*p*<0.05) was adopted for all statistical analyses.

## RESULTS

The results refer to the three stages for selecting the words from the Brazilian Corpus that compose the protocol. In Stage 1, 266 terms were identified, and the following words (176) were chosen: *pé, fé, má, pá, pó, dó, xô, vô, ata, asa, ela, era, ilha, ira, até, axé, aqui, ali, sopa, zona, torá, sofá, cura, pura, guru, tutu, casaca, salada, cilada, pirata, sucata, mulata, careca, tarefa, fivela, tigela, capeta, tabela, cabina, vacina, birita, visita, sulina, tulipa, papuda, maluca, sisuda, sinuca, butuca, sutura, sarará, guaraná, cafuné, canapé, chaminé, picolé, caratê, matinê, jabuti, javali, sururu, jururu, camelô, bibelô, maracatu, tataravô, prata, breve, brisa, prumo, draga, dreno, tropa, fraco, fruta, frevo, cravo, greve, crime, grosso, braço, prece, prima, bruxa, traça, treze, droga, frete, frota, frevo, grade, creme, grilo, grossa, plano, pluma, flora, clero, clima, cloro, glosa, blefe, blusa, flecha, claro, clube, clone, globo, atraso, mutreta, aplauso, conflito, cobra, templo, abrigo, recruta, atleta, emblema, pobre, dupla, braço, glacê, flexão, brechó, clichê, glutão, testa, mesmo, gosto, carta, verde, curto, delta, culto, santo, mente, pasta, misto, custo, perto, circo, morto, calda, filme, conta, mundo, cascata, custoso, mercado, virtude, palmito, soldado, cantada, pintura, construção, circunstância, interstício, baile, noite, azeitona, cuidado, pestana, mistério, partida, curtido, beldade, cultura, mentira, zumbido, menstruação, instrumento, superstição, peito, muito, caiçara, coitado*.

Next, after excluding items with similar syllabic structure and sound, along with the inclusion of terms for phonological similarity pairing for the Repetition and RA lists, 176 words remained. These words and their respective occurrences in Portuguese are presented in Chart 1. In this table, one can also observe the first attempt to distribute the words in the RA and Repetition lists, according to the criteria described for Stage 2.

**Chart 1:** Selected words and their respective occurrences according to the task

**Repetition**	**Occurance of the Repetition list**	**Reading Aloud**	**Occurance of the Reading Aloud list**
*Pé*	38049	*Fé*	22716
*Má*	20768	*Pá*	1342
*Pó*	13574	*Dó*	1472
*Xô*	45	*Vô*	472
*Ata*	12871	*Asa*	2860
*Ela*	348604	*Era*	565796
*Ilha*	19951	*Ira*	3000
*Até*	793522	*Axé*	1944
*Aqui*	210311	*Ali*	48687
*Sopa*	5053	*Zona*	109969
*Torá*	9	*Sofá*	2702
*Cura*	16821	*Pura*	18270
*Guru*	1527	*Tutu*	231
*Casaca*	466	*Salada*	2781
*Cilada*	439	*Pirata*	1493
*Sucata*	1364	*Mulata*	1518
*Careca*	1189	*Tarefa*	56857
*Fivela*	145	*Tigela*	556
*Capeta*	234	*Tabela*	43120
*Cabina*	215	*Vacina*	17051
*Birita*	29	*Visita*	45763
*Sulina*	254	*Tulipa*	127
*Papuda*	6	*Maluca*	828
*Sisuda*	133	*Sinuca*	634
*Butuca*	7	*Sutura*	6403
*Sarará*	36	*Guaraná*	1776
*Cafuné*	150	*Canapé*	106
*Chaminé*	720	*Picolé*	268
*Caratê*	504	*Matinê*	311
*Jabuti*	215	*Javali*	512
*Sururu*	124	*Jururu*	45
*Camelô*	1432	*Bibelô*	81
*Maracatu*	1103	*Tataravô*	95
*Prata*	10477	*Braço*	18301
*Breve*	26394	*Prece*	704
*Brisa*	844	*Prima*	5269
*Prumo*	338	*Bruxa*	1237
*Draga*	160	*Traça*	2984
*Dreno*	1297	*Treze*	4044
*Tropa*	6774	*Droga*	26187
*Fraco*	11926	*Frete*	2124
*Frevo*	1020	*Friso*	316
*Frota*	7580	*Fruta*	5353
*Cravo*	1066	*Grade*	10203
*Greve*	43934	*Creme*	4476
*Crime*	71922	*Grilo*	157
*Grosso*	6432	*Grossa*	3103
*Plano*	119678	*Blefe*	413
*Pluma*	584	*Blusa*	1256
*Flora*	6867	*Flecha*	1474
*Clero*	2869	*Claro*	79646
*Clima*	42357	*Clube*	64253
*Cloro*	3143	*Clone*	3717
*Glosa*	163	*Globo*	4225
*Atraso*	28305	*Abrigo*	7107
*Mutreta*	121	*Recruta*	874
*Aplauso*	1861	*Atleta*	16665
*Conflito*	41289	*Emblema*	1220
*Cobra*	10792	*Pobre*	27368
*Templo*	5731	*Dupla*	34280
*Braço*	18301	*Brechó*	271
*Glacê*	120	*Clichê*	1201
*Flexão*	3948	*Glutão*	65
*Testa*	5594	*Pasta*	12642
*Mesmo*	728088	*Misto*	7544
*Gosto*	31031	*Custo*	103318
*Carta*	40250	*Perto*	44726
*Verde*	24088	*Circo*	5798
*Curto*	40397	*Morto*	33908
*Delta*	2069	*Calda*	2195
*Culto*	9799	*Filme*	124300
*Santo*	6341	*Conta*	201774
*Mente*	25284	*Mundo*	343907
*Cascata*	3140	*Pestana*	17
*Custoso*	529	*Mistério*	7661
*Mercado*	321097	*Partida*	85136
*Virtude*	28026	*Curtido*	394
*Palmito*	2294	*Beldade*	98
*Soldado*	10132	*Cultura*	174245
*Cantada*	1854	*Mentira*	7436
*Pintura*	15739	*Zumbido*	1332
*Construção*	168508	*Menstruação*	2282
*Circunstância*	6046	*Instrumento*	64063
*Interstício*	1006	*Superstição*	1019
*Baile*	3981	*Peito*	13483
*Noite*	116318	*Muito*	681389
*Azeitona*	331	*Caiçara*	226
*Cuidado*	44844	*Coitado*	1048

After that, the word frequencies were studied in quartiles (Table 1). In the quartile analysis, a significant similarity between the lists was observed regarding summary measures. To test this similarity, Spearman’s rank correlation coefficient was used, which yielded a statistically significant correlation between the Repetition and RA lists (*ρ*=0.34; *p*< 0.001).

**Table 1:** Analysis of word occurrence quartiles per list

**Frequency**	**Repetition (N=88)**	**Reading Aloud (N=88)**
Minimum	6	17
1^st^ Quartile	494.5	797
Median	3964.5	2922
Mean	40942.6	36457
3^rd^ Quartile	21598	19405
Maximum	793522	681389

Regarding the classification by syllables, both lists have the same quantity, that is, 04 monosyllabic words, 47 disyllabic words, 33 trisyllabic words, and 04 polysyllabic words.

In addition to the tasks of repetition and RA with controlled words regarding the described variables, the protocol includes tasks of spontaneous conversation, storytelling based on a picture board (bank robbery, taken from the MTL-BR Battery), and the production of the following diadochokinetic syllables: /pa/, /ta/, /ca/, and /pataca/. These tasks are considered the gold standard for the assessment of AOS.

**Figure 1:**
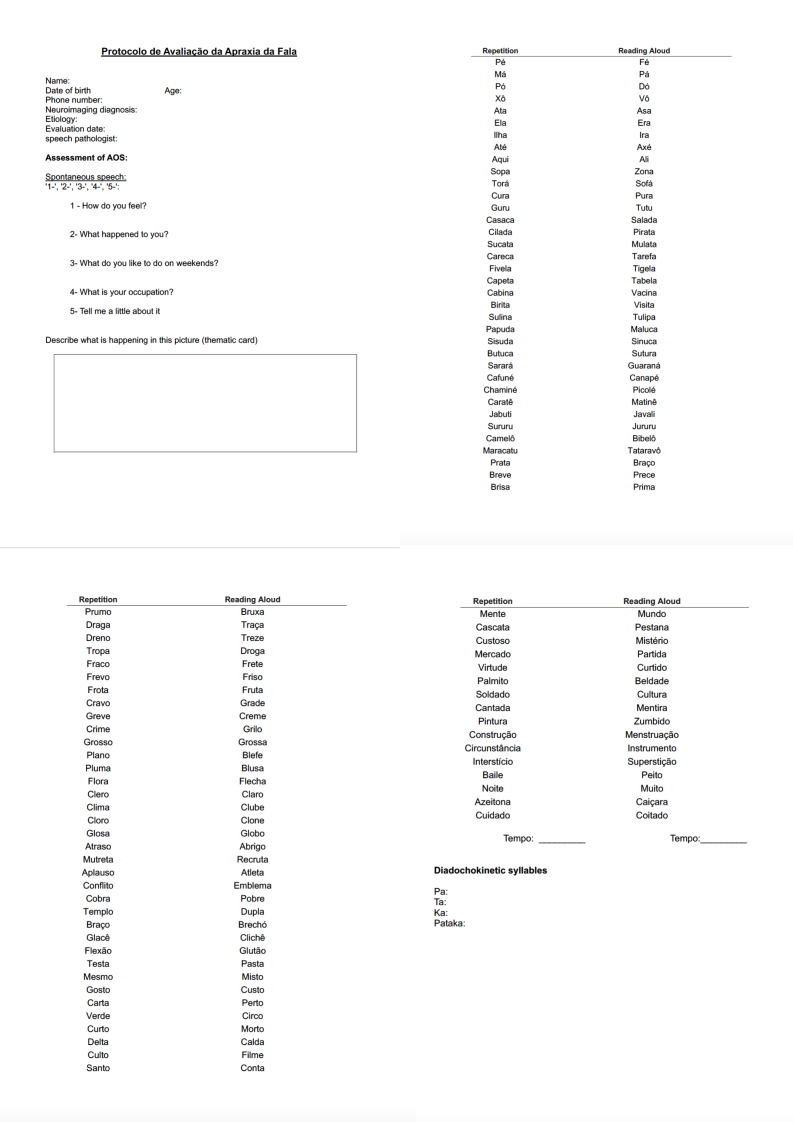
AOS protocol registration sheet

In this stage, a response recording sheet was generated, titled Registration Sheet, which includes participant identification data, stimulus lists, and space for marking correct or incorrect responses, as well as speech production time, considering the variables of articulatory complexity and word frequency in the language. The recording sheet is illustrated in Figure 1. In this regard, this protocol will still be further tested, and it is currently a draft for the pilot study.

## DISCUSSION

The protocol developed for Brazilian Portuguese includes tasks that are considered benchmarks in the international literature for the clinical assessment of AOS.

AOS is a challenging condition to diagnose since it rarely appears in isolation. When it co-occurs with aphasia, which is commonly observed, it is to differentiate between patterns of phonological and phonetic errors through the analysis of speech errors. In this sense, a protocol that controls linguistic variables that interfere with speech-motor production is extremely important. However, even with such a protocol, limitations remain. Indeed, there is currently no internationally recognized assessment methodology or gold standard protocol for diagnosing this speech disorder. Similarly, the classification and analysis of speech errors performed by clinical speech-language pathologists still prove to be difficult and show low interrater agreement^(12)^.

The variables in the word lists were well controlled, focusing on aspects that have the greatest impact on speech motor production, to differentiate between phonetic (apraxia) and phonological (aphasia) errors. It is worth noting that the differential diagnosis between AOS and phonemic paraphasia resulting from phonological impairment due to aphasia should occur through error analysis. However, if the stimuli are not linguistically controlled, this analysis can be challenging or even biased.

Patients with AOS typically exhibit slow and prolonged production of vowels and consonants. Therefore, it is expected that the production time in RA and Repetition tasks will be longer for these individuals compared with that of healthy people^(1,2,5,13-15)^.

The presence of words with various syllabic structures enriches the evaluation, as it is known that individuals with AOS tend to make more errors as the length of words increases^(8)^. Additionally, they experience more difficulty with consonants at the beginning of words^(2,3,9)^ and with less frequently used words in everyday language. Thus, it can be estimated that AOS patients will have poorer performance on longer and less frequent words in the language^(14,15)^. It is also expected that there will be an association between errors in the Repetition and RA lists in these patients.

Advances in the study of speech motor production, with emphasis on perceptual and linguistic aspects, have been valuable in understanding the stages of oral emission. However, while current knowledge about purely phonetic (apraxia) and purely phonological (aphasia) errors assists in the differential diagnosis of these disorders - the large majority of errors found in patients aiming for this differential diagnosis may indeed reflect difficulties in linguistic or motor processing. Therefore, there is still the possibility of difficulty in distinguishing purely motor disorders.

Thus, the developed list will assist in the assessment and therapeutic practice, as it will make the mapping of error types more practical, thereby enabling a more accurate selection of stimuli for each patient.

### Limitations of the study

This is an initial study presenting an assessment protocol for AOS in Brazilian Portuguese with control of linguistic variables and all the necessary tasks for this diagnosis. The variable phonotactic probability was not controlled because there is not sufficient research on this theme in Brazil. Speech samples from healthy individuals should still be compared with those AOS patients to determine if there is a need for changes in the stimuli used in creating this protocol, as well as in the system of error identification, scoring, and establishment of population cutoff scores.

## CONCLUSION

The developed protocol contains tasks considered the gold standard for the assessment of AOS according to the international literature, which makes this instrument highly relevant for diagnosing this disorder in speakers of Brazilian Portuguese.
