# Supplementary material for: Assessment protocol for acquired apraxia of speech
Source: Codas. 2023 Oct 13;36(1):e20220251. doi: 10.1590/2317-1782/20232022251en (PMC10715582; doi:10.1590/2317-1782/20232022251en)
Supplement: Translation

# Translation: Protocolo de avaliação para apraxia de fala adquirida

**DOI:** 10.1590/2317-1782/20232022251pt

**Authors:** Beatriz Maurer Costa, Cláudia Regina Brescancini, Karin Zazo Ortiz

**Affiliations:** 1 Departamento de Fonoaudiologia, Escola Paulista de Medicina, Universidade Federal de São Paulo - UNIFESP - São Paulo (SP), Brasil.; 2 Programa de Pós-graduação em Letras, Escola de Humanidades, Pontifícia Universidade Católica do Rio Grande do Sul - PUCRS - Porto Alegre (RS), Brasil.

**Keywords:** Apraxias, Transtornos da Articulação, Protocolo, Avaliação de Sintomas, Diagnóstico

## Abstract

**Objetivo:**

Elaborar um protocolo de avaliação do planejamento motor da fala com estímulos fonologicamente balanceados para o português brasileiro e que contemple todas as variáveis necessárias para este diagnóstico.

**Método:**

Foram realizadas três etapas: Na primeira, construíram-se listas de palavras cujo critério principal foram os padrões silábicos e acentuais. Do levantamento realizado na Etapa 1, procedeu-se à seleção dos vocábulos que compuseram a primeira versão do protocolo na Etapa 2, reunidas em duas tarefas: de repetição e de Leitura em Voz Alta (LVA). Em seguida, investigou-se a ocorrência das palavras usando a base de dados do Corpus Brasileiro (PUC-SP) - Linguateca. Na etapa 3 realizou-se a análise estatística para verificar se as listas de repetição e de LVA estavam equilibradas quanto à ocorrência das palavras. Assim, as listas foram distribuídas em quartis e foram analisadas de forma descritiva e bivariada. O nível de significância utilizado foi de 5%.

**Resultados:**

Após a realização de todas as etapas, foi possível obter as palavras que compuseram as listas das tarefas de repetição e de LVA. Finalmente, foram então acrescidas ao protocolo as demais tarefas consideradas essenciais para a avaliação da apraxia como as taxas diadococinéticas e a prancha para a emissão oral espontânea.

**Conclusão:**

O protocolo desenvolvido contém as tarefas consideradas padrão para a avaliação da apraxia de fala pela literatura internacional, o que torna esse instrumento importante para o diagnóstico desse distúrbio em falantes do português brasileiro.

## INTRODUÇÃO

A apraxia de fala adquirida foi descrita como “[...] um distúrbio da programação motora da fala manifestada primariamente por erros de articulação”^(1)^. Pode ser decorrente de diversos insultos neurológicos como acidente vascular encefálico, traumatismo cranioencefálico, tumores que invadem o sistema nervoso central e doenças neurodegenerativas. A apraxia de fala costuma acompanhar quadros de afasia^(2)^, o que dificulta a diferenciação das manifestações fonológicas (afasia) das fonéticas(apraxia).

Dentre as características da apraxia de fala, há a repetição de fonemas e de sílabas, a autocorreção e o ensaio articulatório. Manifestações como prolongamento de vogais, aumento da distância interssilábica, distorção fonêmica e chuá intrusivo estão mais diretamente relacionadas à apraxia de fala^(2)^. A substituição, a omissão e a adição, comuns na afasia de condução, também podem ser observadas nos pacientes apráxicos^(2)^. A apraxia também pode ser caracterizada na prosódia por uma fala mais lenta e hesitante com alteração no ritmo e na entonação em virtude de questões do planejamento articulatório^(2,3)^.

Além da descrição de manifestações, é possível e recomendado utilizar modelos linguísticos de produção de fala para análise dos tipos de erros para entender os processos envolvidos nos distúrbios de fala e assim melhor caracterizá-los. O modelo de produção da fala mais aceito atualmente^(4)^ identifica quatro níveis de processamento: o primeiro nível é o pré-motor, também chamado de planejamento linguístico-simbólico, no qual ocorre a seleção dos fonemas e a aplicação das regras linguísticas; o segundo nível é o do planejamento motor, que é responsável pelas memórias motoras e ordenação têmporo-espacial dos fonemas a serem emitidos; no terceiro nível, da programação motora, ocorre a seleção de músculos na sequência apropriada para a produção dos fonemas selecionados; no quarto nível, há a execução da sequência de fala produzida pelos articuladores envolvidos na ação.

De acordo com esse modelo, os sinais de afasia aparecem como um déficit na etapa de planejamento linguístico-simbólico, enquanto os de apraxia aparecem na etapa de planejamento e de programação motora.

Algumas características são típicas e outras exclusivas da apraxia de fala. Distorção de sons, prolongamento de vogais e consoantes e prolongamento de duração de intersegmentos são exclusivas da apraxia de fala^(2,3)^. Tais características são mais evidentes em palavras polissilábicas^(5)^. Mesmo com a publicação do modelo de processamento de fala e a observação de características relacionadas ao planejamento motor, a diferenciação entre erros fonológicos e fonéticos é uma tarefa bastante complexa^(2,6)^.

Com essa mudança na visão e na interpretação da interface fala e linguagem a partir dos modelos de processamento de fala, novos protocolos foram desenvolvidos, tentando tornar o processo de avaliação mais eficaz para a diferenciação de transtornos da linguagem (afasia) e os de fala (apraxia).

No âmbito internacional, destaca-se o *Apraxia Battery for Adults*, ABA-2^(7)^, que possui tarefas com listas de palavras para avaliar a presença e mensurar a gravidade da apraxia de fala do paciente. Isso é feito por meio do *Errors on Words of Increasing Length* (E_WIL), medida que relaciona o erro com a extensão da palavra^(2)^. O *Apraxia of Speech Rating Scale (*ASRS) é uma escala perceptual que, assim como o ABA-2, baseia-se na percepção auditiva do avaliador^(8)^. Nestes protocolos, destaca-se o controle acurado dos estímulos utilizados com as variáveis atualmente consideradas fundamentais para o diagnóstico da apraxia adquirida de fala, incluindo o balanceamento fonológico. Como a análise é subjetiva, existem protocolos que objetivaram reduzir a subjetividade, tais como o *Word Syllable Duration (*WSD), em que se calcula o tempo de produção da palavra dividido pelo número de sílabas produzidas por meio do uso de medidas acústicas^(9)^ e o *Pairwise Variability Index* (PVI), que usa palavras, frases e sentenças para calcular a diferença entre sílabas tônicas e átonas pela duração, frequência fundamental e intensidade^(10)^. Ainda assim, todos os protocolos *contêm* estímulos linguísticos com as variáveis que interferem no planejamento motor como frequência e estrutura das sílabas, posição da sílaba tônica da palavra, balanceamento fonológico, frequência e extensão das palavras e probabilidade fonotática^(2,3,5,8)^. Além disso, preconiza-se o uso de tarefas de emissão espontânea e taxas diadococinéticas^(8)^.

No Brasil há uma escassez de procedimentos objetivos destinados à avaliação clínica da apraxia adquirida de fala. O único protocolo publicado para a avaliação clínica desse quadro^(11)^ prescinde do controle das variáveis linguísticas necessárias para uma acurada avaliação do planejamento motor, bem como de tarefas atualmente consideradas fundamentais para esse diagnóstico.

Diante da mudança na compreensão sobre a produção motora da fala, oriunda dos novos modelos de processamento e a presença de novos instrumentos, surge a necessidade de desenvolver um protocolo para o português brasileiro que contemple as variáveis que interferem no planejamento e na produção motora da fala.

Desse modo, o objetivo desse estudo foi elaborar um protocolo de avaliação para apraxia de fala adquirida com as variáveis importantes para o diagnóstico e com estímulos fonologicamente balanceados para o português brasileiro.

## MÉTODO

Trata-se de um estudo realizado no Departamento de Fonoaudiologia da Escola Paulista de Medicina (EPM), da Universidade Federal de São Paulo.

Para a elaboração do protocolo foram seguidas três etapas:

Na primeira etapa, construíram-se listas de palavras em que foram consideradas palavras de conteúdo, especificamente, substantivos e adjetivos. Além dos substantivos próprios, foram excluídos verbos flexionados ou no infinitivo, palavras funcionais, palavras com contexto para epêntese e palavras cujo significado fosse especializado. Nessa etapa, o critério principal para a construção das listas foram os padrões silábicos e acentuais. Foram consideradas palavras que contemplassem os padrões silábicos da língua portuguesa, do canônico CV ao mais complexo CCVCC, com alternância acentual. Palavras dissílabas e trissílabas formadas somente pelo padrão silábico CV, a saber, CV.CV e CV.CV.CV, foram contempladas em todas as posições acentuais.

Também foram contemplados os seguintes padrões silábicos: CCV em posição tônica em início de palavra; em posição tônica e átona no meio de palavra; CVC em sílaba tônica e átona; CVCC em sílaba átona para coda /lS/ e /RS/ e tônica para /NS/; CVCC em sílaba tônica e átona e CVG (consoante vogal glide) em sílaba átona e tônica, a partir dos ditongos fonológicos /ai/, /ei/, /oi/ e /ui/.

A partir do levantamento realizado na Etapa 1, procedeu-se à seleção dos vocábulos que, de fato, compuseram uma primeira versão da lista. Uma segunda etapa foi necessária em virtude do número excessivo de vocábulos, o que inviabilizaria a aplicação do protocolo. Também foi considerada a necessidade de o protocolo estar composto por palavras com o mesmo tipo de complexidade nas duas tarefas emissivas: repetição e leitura em voz alta. Para isso, foi realizada uma distribuição controlada dos itens entre as duas tarefas, de tal modo que, consideradas todas as vogais orais de todos os itens do protocolo, as palavras da Tarefa de Repetição e de Leitura em Voz Alta tivessem exatamente a mesma proporção de cada uma delas. O mesmo ocorreu para a distribuição das palavras em relação aos fonemas surdos e sonoros que foram controlados.

Durante esta distribuição, para que as listas ficassem idênticas quanto às variáveis pré-determinadas, seria necessário acrescentar três palavras, que seriam correspondentes às que possuem as codas **ls** e **rs**, a saber, “so**ls**tício” e “pe**rs**picaz”, e à que possui o ataque complexo **fl**, especificamente para a palavra “supér**fl**uo”. Para a sílaba CV/lS/, só havia a palavra “so**ls**tício” no português, dados os critérios adotados. Como não há um correspondente em termos de complexidade articulatória para as duas tarefas, essa palavra foi excluída.

Para a sílaba CV/RS/, os substantivos encontrados na língua foram “perspectiva”, “perscrutação”, “superstição” e “interstício”. Foram escolhidas para permanecer na lista as palavras “superstição” e “interstício”.

Por sua vez, a palavra “supérfluo”, presente na primeira lista de palavras devido ao ataque complexo em sílaba átona (pós-tônica), ficou sem um correspondente na Lista de Leitura em Voz Alta porque a única ocorrência que há nesse padrão é a palavra “setênfluo”, cuja frequência nem é registrada no banco do Corpus Brasileiro. Considerou-se, então, como alternativa a palavra “confluído”. No entanto, elas não se equipararam quanto à ocorrência. Por este motivo, esta palavra não foi incluída na lista.

A complexidade do padrão silábico e acentual e as variáveis fonéticas e fonológicas foram controladas pois é a partir delas que é possível realizar uma análise acurada dos erros, incluindo-se, inclusive, a análise da prosódia, em seu aspecto ênfase e a duração das palavras pode também corroborar para a análise da velocidade de produção de fala.

Além dos critérios referentes à complexidade do padrão silábico e acentual e das variáveis fonéticas e fonológicas, houve a necessidade de realização de uma terceira etapa, para o controle da variável frequência das palavras. A seleção dos vocábulos nesta etapa tomou como referência o número de ocorrências verificado no Corpus Brasileiro (PUC-SP), por meio da ferramenta Linguateca. Para isso, todas as ocorrências das palavras pré-selecionadas nas etapas I e II foram identificadas e, posteriormente, analisadas estatisticamente por quartis. Assim, as listas foram analisadas de forma descritiva (por meio do cálculo de medidas resumo) e bivariada pelo coeficiente de correlação de Spearman (rho). Todas as análises foram realizadas no software estatístico R, versão 4.2.1. O nível de significância utilizado foi de 5%.

## RESULTADOS

Os resultados referem-se às etapas para a escolha das palavras que compõem o protocolo. Na primeira etapa foram levantados 266 vocábulos. Os vocábulos escolhidos foram: pé, fé, má, pá, pó, dó, xô, vô, ata, asa, ela, era, ilha, ira, até, axé, aqui, ali, sopa, zona, torá, sofá, cura, pura, guru, tutu, casaca, salada, cilada, pirata, sucata, mulata, careca, tarefa, fivela, tigela, capeta, tabela, cabina, vacina, birita, visita, sulina, tulipa, papuda, maluca, sisuda, sinuca, butuca, sutura, sarará, guaraná, cafuné, canapé, chaminé, picolé, caratê, matinê, jabuti, javali, sururu, jururu, camelô, bibelô, maracatu, tataravô, prata, breve, brisa, prumo, draga, dreno, tropa, fraco, fruta, frevo, cravo, greve, crime, grosso, braço, prece, prima, bruxa, traça, treze, droga, frete, frota, frevo, grade, creme, grilo, grossa, plano, pluma, flora, clero, clima, cloro, glosa, blefe, blusa, flecha, claro, clube, clone, globo, atraso, mutreta, aplauso, conflito, cobra, templo, abrigo, recruta, atleta, emblema, pobre, dupla, braço, glacê, flexão, brechó, clichê, glutão, testa, mesmo, gosto, carta, verde, curto, delta, culto, santo, mente, pasta, misto, custo, perto, circo, morto, calda, filme, conta, mundo, cascata, custoso, mercado, virtude, palmito, soldado, cantada, pintura, construção, circunstância, interstício, baile, noite, azeitona, cuidado, pestana, mistério, partida, curtido, beldade, cultura, mentira, zumbido, menstruação, instrumento, superstição, peito, muito, caiçara, coitado.

Em seguida, com a exclusão de itens com estrutura silábica e sonoridade semelhantes, juntamente com a inclusão de palavras para pareamento em relação à semelhança fonológica para as listas de Repetição e de Leitura em Voz Alta, chegou-se à 176 itens. Os itens e suas respectivas ocorrências na Língua Portuguesa são apresentados no Quadro 1. Nesse quadro, também se pode observar a primeira tentativa de distribuição das palavras nas listas de Leitura em Voz Alta e Repetição, segundo os critérios descritos para a Etapa II.

**Quadro 1:** Palavras selecionadas e suas respectivas ocorrências segundo a tarefa

**Repetição**	**Ocorrência da lista de repetição**	**Leitura em Voz Alta**	**Ocorrência da lista de leitura em voz alta**
Pé	38049	Fé	22716
Má	20768	Pá	1342
Pó	13574	Dó	1472
Xô	45	Vô	472
Ata	12871	Asa	2860
Ela	348604	Era	565796
Ilha	19951	Ira	3000
Até	793522	Axé	1944
Aqui	210311	Ali	48687
Sopa	5053	Zona	109969
Torá	9	Sofá	2702
Cura	16821	Pura	18270
Guru	1527	Tutu	231
Casaca	466	Salada	2781
Cilada	439	Pirata	1493
Sucata	1364	Mulata	1518
Careca	1189	Tarefa	56857
Fivela	145	Tigela	556
Capeta	234	Tabela	43120
Cabina	215	Vacina	17051
Birita	29	Visita	45763
Sulina	254	Tulipa	127
Papuda	6	Maluca	828
Sisuda	133	Sinuca	634
Butuca	7	Sutura	6403
Sarará	36	Guaraná	1776
Cafuné	150	Canapé	106
Chaminé	720	Picolé	268
Caratê	504	Matinê	311
Jabuti	215	Javali	512
Sururu	124	Jururu	45
Camelô	1432	Bibelô	81
Maracatu	1103	Tataravô	95
Prata	10477	Braço	18301
Breve	26394	Prece	704
Brisa	844	Prima	5269
Prumo	338	Bruxa	1237
Draga	160	Traça	2984
Dreno	1297	Treze	4044
Tropa	6774	Droga	26187
Fraco	11926	Frete	2124
Frevo	1020	Friso	316
Frota	7580	Fruta	5353
Cravo	1066	Grade	10203
Greve	43934	Creme	4476
Crime	71922	Grilo	157
Grosso	6432	Grossa	3103
Plano	119678	Blefe	413
Pluma	584	Blusa	1256
Flora	6867	Flecha	1474
Clero	2869	Claro	79646
Clima	42357	Clube	64253
Cloro	3143	Clone	3717
Glosa	163	Globo	4225
Atraso	28305	Abrigo	7107
Mutreta	121	Recruta	874
Aplauso	1861	Atleta	16665
Conflito	41289	Emblema	1220
Cobra	10792	Pobre	27368
Templo	5731	Dupla	34280
Braço	18301	Brechó	271
Glacê	120	Clichê	1201
Flexão	3948	Glutão	65
Testa	5594	Pasta	12642
Mesmo	728088	Misto	7544
Gosto	31031	Custo	103318
Carta	40250	Perto	44726
Verde	24088	Circo	5798
Curto	40397	Morto	33908
Delta	2069	Calda	2195
Culto	9799	Filme	124300
Santo	6341	Conta	201774
Mente	25284	Mundo	343907
Cascata	3140	Pestana	17
Custoso	529	Mistério	7661
Mercado	321097	Partida	85136
Virtude	28026	Curtido	394
Palmito	2294	Beldade	98
Soldado	10132	Cultura	174245
Cantada	1854	Mentira	7436
Pintura	15739	Zumbido	1332
Construção	168508	Menstruação	2282
Circunstância	6046	Instrumento	64063
Interstício	1006	Superstição	1019
Baile	3981	Peito	13483
Noite	116318	Muito	681389
Azeitona	331	Caiçara	226
Cuidado	44844	Coitado	1048

Em seguida, realizou-se o estudo das frequências das palavras em quartis (Tabela 1). Na análise dos quartis, observa-se grande semelhança entre as listas quanto as medidas resumo. Para testar essa semelhança, utilizou-se a correlação de Spearman em que se obteve correlação estatisticamente significante entre as listas de Repetição e Leitura em Voz Alta (rho = 0,34, p-valor < 0,001).

**Tabela 1:** Análise dos quartis de ocorrências de palavras por lista

**Frequência**	**Repetição (N=88)**	**Leitura Em Voz Alta (N=88)**
Mínimo	6	17
1° Quartil	494.5	797
Mediana	3964.5	2922
Média	40942.6	36457
3° Quartil	21598	19405
Máximo	793522	681389

Quanto à classificação por sílabas, ambas as listas apresentam a mesma quantidade, isto é, 04 monossílabos, 47 dissílabos, 33 trissílabos e 04 polissílabos.

Além das tarefas de repetição e leitura em voz alta com vocábulos controlados quanto às variáveis descritas, o protocolo conta com as tarefas de conversa espontânea, emissão de história a partir de uma prancha de figura (assalto ao banco, retiradas da Bateria MTL-BR) e emissão das diadococinesias /pa/, /ta/, /ca/ e /pataca/. Tais tarefas são consideradas padrão ouro para a avaliação da apraxia de fala adquirida.

Nessa etapa foi gerada a folha para marcação das respostas, intitulada “Folha de Registro”, a qual conta com os dados de identificação do participante, lista dos estímulos e espaço para a marcação da emissão correta ou não, e tempo de produção da fala, consideradas as variáveis complexidade articulatória e frequência da palavra na língua. A folha de marcação é apresentada na Figura 1. Nesse sentido, esse protocolo ainda será testado, sendo um esboço para o estudo piloto.

**Figura 1:** Folha de respostas do protocolo de apraxia adquirida

## DISCUSSÃO

O protocolo desenvolvido para o português brasileiro conta com tarefas que são referências na literatura internacional para a avaliação clínica da apraxia de fala adquirida.

A apraxia de fala adquirida é um quadro difícil de ser diagnosticado, uma vez que raramente aparece de forma isolada e, quando coocorre com a afasia, o que mais comumente se observa, há necessidade de que, a partir da análise dos erros da fala, seja possível diferenciar padrões de erros fonológicos dos fonéticos. Nesse sentido, um protocolo que controle as variáveis linguísticas que interferem na produção motora da fala é extremamente importante. No entanto, ainda assim, apresenta limitações. De fato, não há ainda na literatura internacional uma metodologia de avaliação ou um protocolo que possa ser considerado padrão-ouro para o diagnóstico deste distúrbio de fala e, da mesma forma, a classificação e a análise dos erros de fala realizada pelos fonoaudiólogos clínicos ainda se mostra difícil e com baixa concordância interavaliadores^(12)^.

As variáveis das listas de palavras foram bem controladas, focando-se nos aspectos que mais interferem na produção motora da fala, a fim de diferenciar claramente os erros de natureza fonética (apraxia) dos erros fonológicos (afasia). Vale lembrar que o diagnóstico diferencial entre apraxia de fala e parafasias fonêmicas oriundas de alteração fonológica devido à afasia deve ser feito pela análise dos erros. No entanto, se os estímulos não estiverem controlados linguisticamente, esta análise pode ser dificultada ou até enviesada.

Como a fala dos pacientes com apraxia de fala adquirida caracteriza-se por lentidão e prolongamento de vogais e consoantes, espera-se que o tempo de produção nas listas de leitura e repetição sejam maiores do que nos indivíduos saudáveis^(1,2,5,13-15)^.

A presença de palavras com várias estruturas silábicas torna mais rica a avaliação, pois sabe-se que os apráxicos tendem a cometer mais erros nas palavras conforme há o aumento de extensão delas^(8)^. Além disso, eles apresentam mais dificuldade em consoantes no início da palavra^(2,3,9)^ e em palavras menos utilizadas no cotidiano. Dessa forma, pode-se estimar que os pacientes com apraxia de fala adquirida terão um pior desempenho nas palavras mais extensas e menos frequentes na língua^(14,15)^. Espera-se também que haja uma associação entre os erros na lista de Repetição e os erros na lista de Leitura em Voz alta nos pacientes com apraxia.

Os avanços no estudo de produção motora da fala, valorizando questões perceptuais e linguísticas, foram valiosos para a compreensão das etapas da emissão oral. No entanto, embora o conhecimento que se tenha hoje sobre erros de natureza puramente fonética (apraxia) e erros puramente fonológicos (afasia) auxilie no diagnóstico diferencial entre estes transtornos, a grande maioria dos erros encontrados em pacientes em que se objetiva esse diagnóstico diferencial, podem, de fato, expressar falhas no processamento linguístico ou motor. Portanto, ainda há a possibilidade de dificuldade na distinção de quadros puramente motores.

Assim, a lista desenvolvida auxiliará na avaliação e na prática terapêutica, uma vez que tornará mais prático o mapeamento dos tipos de erros e, consequentemente, possibilitará uma seleção mais apurada dos estímulos para cada paciente.

### Limitações do estudo

Trata-se de um estudo inicial em que se apresenta um protocolo de avaliação de apraxia de fala para o português brasileiro com o controle das variáveis linguísticas e com todas as tarefas necessárias para esse diagnóstico. A variável probabilidade fonotática não foi controlada porque não há estudos suficientes no Brasil nesta temática. Amostras de fala de indivíduos saudáveis ainda deverão ser comparadas aos de indivíduos com apraxia adquirida de fala, a fim de verificarmos se haverá necessidade de mudanças nos estímulos obtidos na criação deste protocolo, bem como no sistema de identificação de erros, atribuição de escores e obtenção de notas de corte populacionais.

## CONCLUSÃO

O protocolo desenvolvido contém tarefas consideradas padrão ouro para a avaliação da apraxia de fala pela literatura internacional, o que torna esse instrumento de grande relevância para o diagnóstico desse distúrbio em falantes do português brasileiro.
